# Genome sequence comparisons of serial multi-drug-resistant *Mycobacterium tuberculosis* isolates over 21 years of infection in a single patient

**DOI:** 10.1099/mgen.0.000037

**Published:** 2015-11-26

**Authors:** Ella M. Meumann, Maria Globan, Janet A. M. Fyfe, David Leslie, Jessica L. Porter, Torsten Seemann, Justin Denholm, Timothy P. Stinear

**Affiliations:** ^1^​ Victorian Infectious Disease Service, Melbourne Health, Melbourne, Victoria 3000, Australia; ^2^​ Doherty Institute for Infection and Immunity, Victoria 3000, Australia; ^3^​ Mycobacterium Reference Laboratory, Victorian Infectious Diseases Reference Laboratory, Melbourne Health, Melbourne, Victoria 3000, Australia; ^4^​ Department of Microbiology and Immunology, University of Melbourne, Melbourne, Victoria 3000, Australia; ^5^​ Victorian Life Sciences Computation Initiative, University of Melbourne, Parkville, Victoria 3010, Australia; ^6^​ Doherty Applied Microbial Genomics, Doherty Institute for Infection and Immunity, Melbourne, Victoria 3000, Australia; ^7^​ Victorian Tuberculosis Program, Melbourne, Victoria 3000, Australia

**Keywords:** antibiotic resistance, clinical persistence, evolution, *Mycobacterium tuberculosis*

## Abstract

We report a case of chronic pulmonary multi-drug-resistant tuberculosis. Despite 14 years of treatment, *Mycobacterium tuberculosis* was persistently isolated from sputum. Following treatment cessation the patient remained well, although *M. tuberculosis* was isolated from sputum for a further 8 years. Genome sequencing of eight serial *M. tuberculosis* isolates cultured between 1991 and 2011 revealed 17 mutations (0.8 mutations per genome year^− 1^). Eight of these were persisting mutations and only two mutations were detected in the 7 years following cessation of treatment in 2004. In four isolates there were mixed alleles, suggesting the likely presence of bacterial subpopulations. The initial 1991 isolate demonstrated genotypic resistance to isoniazid (*katG* W91R), rifampicin (*rpoB* S531L), ethambutol (*embB* M306V), streptomycin (*gidB* L16R), quinolones (*gyrA* S95T) and P-aminosalicylic acid (*thyA* T202A). Subsequent resistance mutations developed for pyrazinamide (*pncA* I31F) and ethionamide (*ethA* frameshift). Such information might have been instructive when developing a treatment regimen. In retrospect and with the benefit of high-resolution genomic hindsight we were able to determine that the patient received only one or two active anti-tuberculous agents for most of their treatment. Additionally, mutations in *bacA* and Rv2326c were detected, which may have contributed to the persistent but mild disease course. BacA is likely to be associated with maintenance of chronic infection and Rv2326c with a decreased bacterial metabolic state. These results expand our understanding of *M. tuberculosis* evolution during human infection and underline the link between antibiotic resistance and clinical persistence.

## Data Summary

1. Supplementary Table S1 is available through Fihare. http://dx.doi.org/10.6084/m9.figshare.1561411
2. Sequence read data for the study isolates have been submitted to the EMBL Sequence Read Archive (ENA). https://www.ebi.ac.uk/ena/data/view/ERP011175

## Impact Statement

Tuberculosis (TB) is a major cause of human disease globally. The causative bacterium, *Mycobacterium tuberculosis*, has evolved with humans for many thousands of years. TB has wide-ranging disease manifestations. Factors related to the human host and the state of the immune system are well known to influence the disease progression of TB, while factors relating to the bacterium itself have been relatively elusive. Whole genome sequencing involves unravelling the entire sequence of bacterial DNA, and technology to do this has become increasingly available. Here we describe an unusual case of multi-drug-resistant TB, and present the results of whole genome sequencing of serial isolates collected over a 21-year period. This has enabled us to further our understanding of the evolution of the TB bacterium within the human host. This gives us insight into the development of drug resistance mutations and other mutations that may alter the disease course, and ultimately contributes to increased understanding of the biology of this important human disease.

## Introduction

Infection with *Mycobacterium tuberculosis* is a major cause of human morbidity and mortality, and has wide-ranging clinical manifestations. While host factors, particularly T-cell-mediated immunity, are well known to influence disease presentation and outcome, bacterial factors are also important, but elusive to pinpoint. The majority of studies assessing strain-specific factors have looked at associations between lineage and disease manifestation; at present there is little known about the role of specific genes in determining disease phenotype (Coscolla & Gagneux, 2010). In recent years, technology allowing large-scale whole genome sequencing (WGS) has become increasingly available, which is facilitating a rapid increase in our understanding of the evolution of bacterial populations and in particular molecular mechanisms of drug resistance.

Here we have used genomics to investigate the evolution of *M. tuberculosis* during human pulmonary infection. We describe a case of chronic pulmonary multi-drug-resistant tuberculosis (TB), and report the results of WGS analysis of eight serial *M. tuberculosis* isolates collected from the same patient over a 21-year period. We describe the heterogeneity in sequencing results suggestive of dynamic bacterial subpopulations, and provide an estimate of the mutation rate of *M. tuberculosis* during active infection. We describe the baseline resistance-associated mutations and subsequent mutations that explain the changing pattern of drug resistance, and the potential influence of emergent mutations in the genes encoding putative exporter proteins, *bacA*, Rv1819c, Rv2326c and *mshA*, on the disease course.

## Methods

Primary culture of *M. tuberculosis* from clinical specimens was performed using pyruvate-enriched Lowenstein–Jensen and Brown & Buckle media. Specimens received from 2007 onwards were also cultured within Mycobacteria Growth Indicator Tubes. Primary *M. tuberculosis* culture and susceptibility testing was also performed using the radiometric BACTEC 460TB system (Becton Dickinson) from 1991 to 2007, and from 2007 onwards using the BACTEC MGIT 960 system (Becton Dickinson). DNA was extracted from isolates cultured from sputum specimens as described by Ross *et al.* (1991). Isolate ID numbers and isolation dates are as follows: Mtb_41, 3 January 1991; Mtb_323, 14 May 1992; Mtb_682, 6 October 1993; Mtb_1087, 30 May 1995: Mtb_2573, 6 February 2002; Mtb_2855, 18 June 2003; Mtb_4891, 16 March 2010; Mtb_5288, 7 June 2011.

Whole genome DNA sequencing was performed on an Illumina MiSeq using 250 bp paired-end reads, with library preparation using Nextera XT (Illumina). Prior to mapping, reads were trimmed to remove adaptor sequences and low-quality bases (less than Q10). Reads less than 50 nt in length were removed. Resulting sequence fastq files were aligned to the *M. tuberculosis* H37Rv reference chromosome (NC_000962.3) using Snippy v2.6 (https://github.com/tseemann/snippy) and Nesoni v0.130 (https://github.com/Victorian-Bioinformatics-Consortium/nesoni) to identify single nucleotide polymorphisms (SNPs). Mapping was constrained to non-repetitive sequences, representing 98.4 % coverage of the H37Rv reference. Average depth of coverage per genome was as follows: Mtb_41, 215 × ; Mtb_323, 151 × ; Mtb_682, 38 × ; Mtb_1087, 46 × ; Mtb_2573, 109 × ; Mtb_2855, 34 × ; Mtb_4891, 128 × ; Mtb_5288, 12 × . The low coverage of Mtb_5288 was below our minimum target coverage of 30 ×  but additional DNA from this isolate was unavailable so was included here with the caveat of potential SNP-calling unreliability. Bi-allelic mutations were called when read-mapping indicated the presence of an alternative nucleotide in greater than 10 % of reads across a given position in the reference. The Variant Call Format (VCF) files describing the SNPs for each isolate were uploaded to P (http://pathogenseq.lshtm.ac.uk/phytblive/index.php) to determine the closest of the seven *M. tuberculosis* complex lineages and as an initial screen for mutations linked to antibiotic resistance. Resistance mutations were also identified with reference to the TB Drug Resistance Mutation Database (http://www.tbdreamdb.com/) (Sandgren *et al.*, 2009). IS6110 insertion site mapping was performed with IS_mapper (https://github.com/jhawkey/IS_mapper), using NC_000962.3 as a reference (Hawkey *et al.*, 2015).

## Results and Discussion

### Case history

**Fig. 1.**
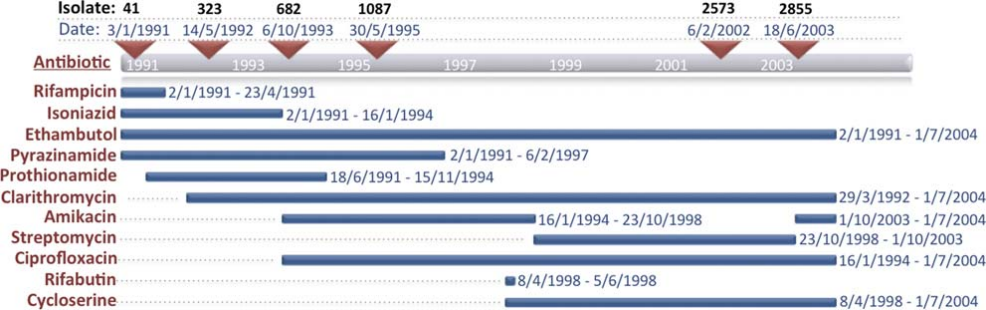
MDR-TB therapy administered, 1991–2004. Depicted in red on the left are the antibiotics used with the date that each treatment was initiated and the duration indicated by the blue horizontal bars. Red triangles indicate the date sputum samples were collected from which *M. tuberculosis* was isolated in culture. The isolate ID numbers are shown above the sample collection date.

**Fig. 2:**
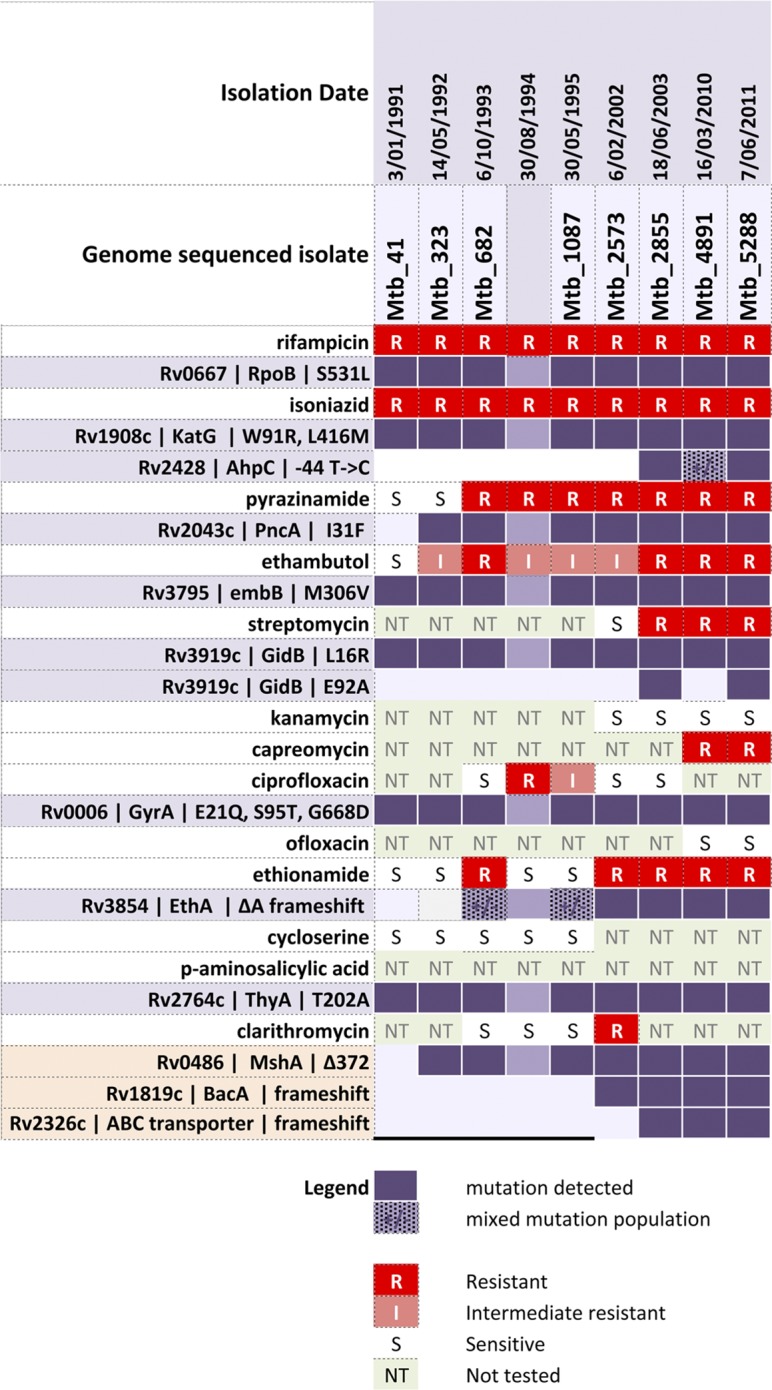
Summary of *M. tuberculosis* mutations occurring over 20 years. The left-hand column shows antibiotic (white rows with red blocks for resistance) and resistance gene/locus implicated (mauve rows with purple blocks indicate presence of the resistance-conferring mutation). Note that a DNA sample was not available for sequencing the isolate from 30 August 1994 although resistance testing was performed.

In January 1991, a 35-year-old East Timorese female presented with haemoptysis, sweats and 5 kg weight loss over 6 months. She had arrived in Melbourne, Australia, in October 1990, and had been treated for pulmonary TB with an unknown treatment regimen during the year leading up to her arrival in Australia. The patient's admission chest radiograph demonstrated left upper lobe consolidation, and sputum was smear-positive for acid-fast bacilli (AFB). She commenced treatment with isoniazid, rifampicin, pyrazinamide and ethambutol for a presumptive diagnosis of TB. *M. tuberculosis* was subsequently isolated from sputum. Multiple changes were made to the treatment regimen as summarized in Fig. 1. Susceptibility testing demonstrated resistance to isoniazid and rifampicin but susceptibility to ethambutol and pyrazinamide, confirming a diagnosis of multidrug-resistant TB (MDR-TB). Susceptibility testing results are summarized in Fig. 2. The patient remained symptomatic with persistent cough and haemoptysis, and remained smear-positive for AFB and culture-positive for *M. tuberculosis*. In April 1992, the patient underwent left upper lobectomy, and excision of the apical segment of the left lower lobe. Following this, the patient's cough resolved. She remained well from that time, although she was able to produce sputum that was intermittently smear-positive for AFB, and persistently culture positive for *M. tuberculosis*. In July 2004, the patient remained well and all medications were stopped, although the preceding sputum specimen had been culture-positive for *M. tuberculosis*. The patient was reviewed regularly between then and 2010 and remained well. She was still well in 2010 when a repeat sputum specimen was smear-negative but culture-positive for *M. tuberculosis*. Treatment was not reinitiated. Subsequent sputum specimens in 2011 and 2012 were culture-positive for *M. tuberculosis* but two sputum specimens in 2014 were culture-negative.

### Initial genotypic multi-drug resistance

Taking advantage of DNA preparations stored by the reference laboratory from initial genotyping analysis, eight *M. tuberculosis* genomes were sequenced spanning the 20 years of the 21-year treatment and observation period. Genome comparisons of the initial 1991 isolate (isolate 41) against a database of 1601 *M. tuberculosis*-complex genome sequences showed that isolate 41 was a lineage 4 strain (lineage 4.3.4.1), and confirmed that it contained mutations in *rpoB* and *katG*, which corresponded with phenotypic resistance to rifampicin and isoniazid (Fig. 2). Isolate 41 also had a mutation in *embB*, which is associated with ethambutol resistance (Sreevatsan *et al.*, 1997b). Initial susceptibility testing in 1991 suggested the isolate was sensitive to this agent. Subsequent isolates demonstrated reduced ethambutol susceptibility.

In addition to the mutations associated with resistance to first-line agents, isolate 41 also harboured mutations associated with resistance to streptomycin, quinolones and *p*-aminosalicylic acid (PAS). This included a mutation in *gidB* linked to streptomycin resistance (Okamoto *et al.*, 2007). Streptomycin susceptibility testing was not done until 1998, at which point the isolate tested susceptible to streptomycin, although after 5 years of treatment with streptomycin phenotypic resistance subsequently developed in 2003. There were no mutations in aminoglycoside-resistance-conferring genes including *rrs*, *rpsL* or *eis* (Zaunbrecher *et al.*, 2009). Isolate 41 had three *gyrA* mutations, one of which (S95T) has been previously reported in association with reduced susceptibility to quinolones (Kapur *et al.*, 1995). Quinolone susceptibility testing was not done until 1993, at which point the isolate tested susceptible to ciprofloxacin. However, subsequent decreased susceptibility to ciprofloxacin was observed in four of 15 isolates during the 10 years of treatment with this agent from 1994 to 2003. Isolate 41 also possessed a characteristic mutation in *thyA* that is associated with resistance to PAS (Rengarajan *et al.*, 2004) (Fig. 2). However, phenotypic PAS susceptibility testing was never undertaken and it is uncertain whether the patient had previously received PAS as part of TB treatment in East Timor.

At the outset of treatment in 1991, the patient was therefore infected with an *M. tuberculosis* strain harbouring genotypic resistance to four first-line drugs (rifampicin, isoniazid, ethambutol and streptomycin) and two second-line drugs (quinolones and PAS). If a genomic approach had been available in 1991, knowledge of this resistance profile at the commencement of treatment may have prevented exposure to unnecessary toxic drugs, and allowed composition of an effective regimen that included enough active drugs to prevent emergence of further resistance. Phenotypic susceptibility testing is known to be unreliable for ethambutol, pyrazinamide and some second-line agents, which may account for the discordant phenotype/genotype results (Hillemann *et al.*, 2013). While the increased availability of WGS has led to a rapid increase in available sequence data, there is significant remaining uncertainty surrounding the phenotypic implications of putative drug resistance mutations. It is also likely that some antibiotic resistance-associated genes are yet to be identified (Witney *et al.*, 2015). A standardized method for large-scale identification of drug resistance mutations has recently been proposed (Walker *et al.*, 2015).

### Additional drug resistance mutations arising during 14 years of treatment

Treatment with pyrazinamide began in January 1991, and pyrazinamide resistance was first detected in June 1993. Genome analysis uncovered a resistance-conferring *pncA* mutation in May 1992, 13 months prior to the first detection of phenotypic resistance (Fig. 2). Prothionamide treatment began in June 1991, with ethionamide resistance observed in October 1993 and the appearance of an ethionamide resistance mutation in *ethA* at that time (Fig. 2). Heterogeneity was initially present; 26 % of sequence reads for the October 1993 isolate demonstrated the resistance-conferring *et* mutation and the remainder were consistent with wild-type (Table S1, available in the online Supplementary Material). This population dynamic subsequently shifted such that the Δ*ethA* mutant was dominant and the wild-type sequence was no longer detected (Fig. 2, Table 1). An additional mutation linked to isoniazid resistance, an intergenic nucleotide substitution 44 bp upstream of *ahpC*, was identified in the June 2003 isolate (Sreevatsan *et al.*, 1997a). Also in June 2003, a second *gidB* mutation (E92A) was identified at the same time that streptomycin resistance was detected. To our knowledge, this mutation has not been previously identified as being associated with streptomycin resistance. We hypothesize that the combination of the existing L16R with the subsequent E92A mutation may have had an additive effect and led to emergence of phenotypic resistance to streptomycin. Of note, there was re-emergence of the wild-type allele in March 2010 but reversion to E92A by June 2011.

**Table 1:**
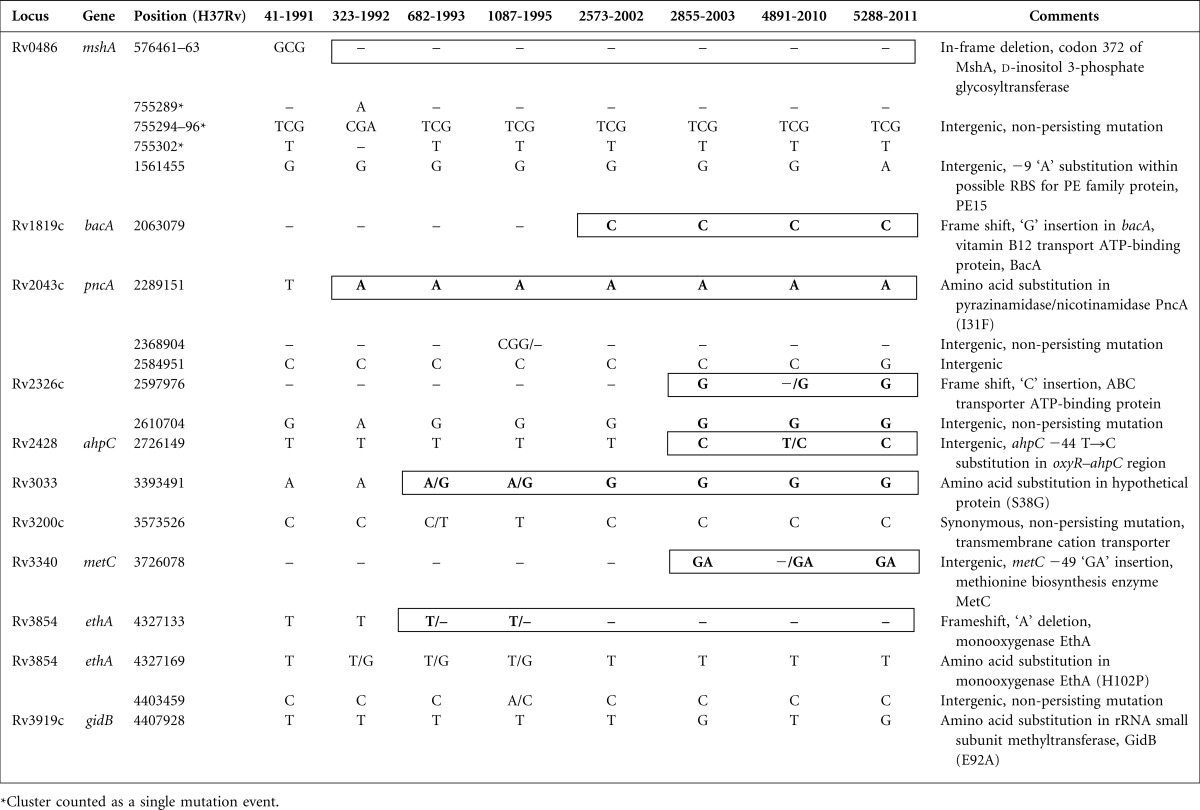
Mutations detected among the seven *M. tuberculosis* isolates over 20 years compared with initial isolate 41–, Deletion; bold type and bold border indicate persisting mutation; ‘X/Y’ indicates bi-allelic base call.

The lag between genotypic and phenotypic resistance and the heterogeneity of sequence reads is likely to be due to the presence of subpopulations of *M. tuberculosis* with different resistance profiles. Heterogeneity in susceptibility results for different *M. tuberculosis* colonies cultured from the same sputum specimen has previously been described (Mariam *et al.*, 2011; Sun *et al.*, 2012). There is evidence to suggest that such heterogeneity is associated with increased likelihood of a poor clinical outcome (Zetola *et al.*, 2014). With this constellation of drug resistance-associated mutations, the patient received a regimen with only one or two active agents for the majority of her 14 years of treatment.

### Very low *M. tuberculosis* mutation rate during active infection

The mutation rate for *M. tuberculosis* compared with other pathogens is low and estimated at 0.3-0.5 mutations per genome year^− 1^ (Ford *et al.*, 2011), and is thought to be similar in latent, active and recently reactivated disease (Ford *et al.*, 2013). Across the eight genomes collected over a 21-year period of active infection, 17 mutations were detected, representing a mutation rate of 0.8 mutations per genome year^− 1^ if all mutations are considered (Tables 1 and S1). The majority of mutations occurred during antibiotic treatment. There were only two new mutations detected between cessation of treatment in 2004 and the final isolate in 2011. We attempted to examine the background mutation rate by counting only synonymous mutations. However, all within-CDS (coding DNA sequence) nucleotide mutations that became fixed in the population over 20 years were also non-synonymous, i.e. they changed the amino acid sequence. Furthermore, the two intergenic SNPs that became fixed (positions 2726149 and 3726078) were probably promoter-associated mutations and so had a functional consequence (Table 1). These observations indicate a mutation rate much lower than previous reports, with no background mutations occurring over 20 years of active infection.

The genome of each isolate harboured eight copies of IS6110 with no insertion site variation between genomes (data not shown). The isolates all harboured an IS6110 copy in the or region between *dnaA* and *dnaN*, as has been described previously by Turcios *et al.* (2009).

### *M. tuberculosis* population heterogeneity

In four isolates there were mixed alleles for some loci, where read-mapping indicated two potential sequence variants at a particular chromosome position (Tables 1 and S1). This observation highlights the interconnected and shifting nature of the bacterial population within the patient, and is consistent with the presence of subpopulations, with dynamic changes occurring as a result of selection pressures such as antibiotic exposure and host immune responses.

### Clinical persistence-associated mutations

In February 2002, a loss-of-function frameshift mutation was identified in *bacA*. This gene encodes a putative membrane protein (BacA) and its orthologues have been implicated in the maintenance of chronic infection and/or symbiosis in *Brucella abortus* (Lier *et al.*, 2000) and *Sinorhizobium melliloti* (Glazebrook *et al.*, 1993) and their respective hosts. In *M. tuberculosis*, *bacA* is thought to encode an ABC transporter. A laboratory-derived *M. tuberculosis bacA* deletion mutant exhibited reduced susceptibility to aminoglycosides and prolonged survival in a murine lung infection model (221 days) compared with wild-type (156 days), indicating a role for BacA in chronicity of infection (Domenech *et al.*, 2009). Interestingly, *bacA* has been reported to be upregulated in the context of rifampicin monoresistance, providing another example of a link between this gene and antibiotic exposure (Li *et al.*, 2015).

Between February 2002 and June 2003, a nucleotide insertion occurred causing a frameshift mutation in Rv2623c, a putative ABC transporter ATP-binding protein (Yousuf *et al.*, 2015). Expression of the transcriptional regulator Rv0494 is increased in *M. tuberculosis* during conditions of starvation such as during dormancy (Yousuf *et al.*, 2015). Furthermore, Rv0494 has been shown to downregulate expression of Rv2623c (Yousuf *et al.*, 2015). This suggests that latency is associated with decreased expression of Rv2623c. We propose that the Rv2623c loss-of-function mutation may reduce *M. tuberculosis* metabolic activity, and that this may have contributed to the patient's relatively indolent disease progression.

An in-frame deletion of codon 372 of Rv0486 *mshA* first occurred in May 1992. MshA is an enzyme required for production of a mycothiol precursor, 1-*O*-(2-acetamido-2-deoxy-α-d-glucopyranosyl)-d-*myo*-inositol (Newton *et al.*, 2006). Mycothiol is a glutathione-like thiol produced by *M. tuberculosis* and other *Actinobacteria*. Mycothiol has an important role in defence against alkylating agents and reactive oxygen and nitrogen species, and is essential for mycobacterial growth (Newton *et al.*, 2008). MshA mutagenesis studies indicate that this enzyme is required for mycobacterial growth and survival (Buchmeier & Fahey, 2006). MshA also has been implicated in drug resistance, arising during patient treatment with ethionamide (Vilchèze *et al.*, 2008). The *mshA* codon-372 deletion may alter bacterial fitness but the impact of this change is uncertain.

An intergenic substitution mutation − 9 bp upstream of Rv1819c was observed in the 2011 isolate. This mutation appears to have altered the ribosome binding site (RBS) for this CDS, changing the motif ‘GGAG’ to ‘GGAA’. Rv1819c endodes a PE/PPE family protein PE15. PE15 has been associated with altered inflammatory immune responses and intracellular survival and we speculate that altered expression of PE15 may also help to explain the persistence of this infection (Tiwari *et al.*, 2012).

The patient initially had severe symptoms of TB and evidence of advanced and progressive pulmonary infection. Her disease burden was reduced significantly with a lobectomy. This procedure was not curative, however, and *M. tuberculosis* persisted thereafter for many years. Despite culture positivity at the time of treatment cessation, the disease did not progress in this patient; she appeared to remain in homeostasis with the organism for many years subsequently. We hypothesize that the mutations in *bacA*, Rv2326c and potentially *mshA* contributed to the persistent but mild disease course in this patient. However, we also recognize that each of these genes has been linked previously in various ways to resistance, and thus the driving pressures for changes in these loci may have been the antibiotics, not host immune pressures per se. Note, however, that in other pathogens resistance mutations can confer persister phenotypes. Studies of clinical *Staphylococcus aureus* show that rifampicin-resistance-conferring mutations in *rpoB* lead to increased resistance to innate immune killing mechanisms and increased *in vivo* persistence (Gao *et al.*, 2013).

It is also likely that the resistance-associated mutations observed in this study have altered bacterial fitness. Resistance mutations have varying effects on the fitness of *M. tuberculosis*. These effects are often moderated by compensatory mutations at other sites (Müller *et al.*, 2013). For example, mutations in *rpoB* are frequently associated with decreased fitness, although the effect can be decreased by compensatory mutations in *rpoB* or *rpoB* (Gagneux *et al.*, 2006). Strains with mutations leading to low or no fitness cost account for the majority of drug-resistant isolates from clinical specimens; there is evidence that these strains are positively selected for over time when heteroresistance is present, and are more easily transmitted than resistant strains with high fitness cost (Müller *et al.*, 2013).

## Conclusion

We describe an unusual case of MDR-TB characterized by treatment failure but an indolent course. Initial phenotypic susceptibility testing revealed resistance to isoniazid and rifampicin, although at treatment outset there was unknown genotypic resistance to these drugs as well as to ethambutol, quinolones, streptomycin and PAS. Subsequent resistance-associated mutations for pyrazinamide and ethionamide emerged. If this genotypic resistance profile is considered reliable, the patient was therefore treated with just one or two active drugs for the majority of the 14-year treatment duration. The lag between genotypic and phenotypic resistance and the heterogeneity of sequence reads are suggestive of bacterial subpopulations with different resistance profiles, although some discordance between genotypic and phenotypic results may be due to unreliable phenotypic susceptibility testing. The majority of mutations identified occurred during the period of multi-drug treatment and were associated with drug resistance. The additional emergence of mutations in *bacA*, RV2326c, Rv1819 and *mshA* may have contributed to the indolent but persistent disease course. Our study follows a previous report that used genomics similarly to describe the evolution of XDR-TB in a single patient over a 4-year period (Eldholm *et al.*, 2014). This report also uncovered considerable within-sample genomic heterogeneity, as well as identifying mutations and expression changes in *M. tuberculosis* export-associated genes that might be linked with increased resistance (Eldholm *et al.*, 2014). WGS of serial *M. tuberculosis* isolates from single patient series are providing important real-world insights into within-host evolution of *M. tuberculosis* during periods with and without treatment. Findings from these studies have implications for treatment but also for aiding interpretation of genome sequence variation in the context of *M. tuberculosis* transmission.

## Supplementary Data

60 Supplementary Data
