# Supplementary material for: Genome sequence comparisons of serial multi-drug-resistant Mycobacterium tuberculosis isolates over 21 years of infection in a single patient
Source: Microb Genom. 2015 Nov 26;1(5):e000037. doi: 10.1099/mgen.0.000037 (PMC5320678; doi:10.1099/mgen.0.000037)
Supplement: Supplementary file 1 — Supplementary Data [file mgen-01-37-s001.pdf]

Supplementary Table 1: Mutations detected among the seven isolates over 20 years compared to 41

				SNP call								Observed read coverage and composition across each position								
**Locus_tag**	**Gene**	**Position**	**Change type**	**41-1991**	**323-1992**	**682-1993**	**1087-1995**	**2573-2002**	**2855-2003**	**4891-2010**	**5288-2011**	**41-1991**	**323-1992**	**682-1993**	**1087-1995**	**2573-2002**	**2855-2003**	**4891-2010**	**5288-2011**	**Comments**
**Rv0486**	***mshA***	576461-63	deletion	GCG	**-**	**-**	**-**	**-**	**-**	**-**	**-**	GCGx216	-x130 Gx5	-x17 Gx1	-x27 Gx1	-x58 Gx2	-x16 Gx1	-x83 Gx4	-x13	In-frame deletion, codon 372 of MshA, D-inositol 3-phosphate glycosyltransferase
		755289*	insertion	-	A	-	-	-	-	-	-	-x217	Ax104 -x19	-x25	-x29 Ax1	-x67	-x18 Ax1	-x90	-x11	
		755294-96*	substitution	TCG	CGA	TCG	TCG	TCG	TCG	TCG	TCG	TCGx215 Cx2	CGAx106 TCGx19	TCGx25	TCGx28 Cx1	TCGx67	TCGx18 Cx1	TCGx88 Cx1	TCGx11	Intergenic, non-persisting mutation
		755302*	deletion	T	-	T	T	T	T	T	T	Tx218 Ax3	-x105 Tx22	Tx26	Tx27 -x1	Tx72	Tx14 -x1	Tx88 -x5	Tx11	
**Rv1386**		1561455	substitution	G	G	G	G	G	G	G	A	Gx161	Gx77	Gx19	Gx18	Gx77	Gx12	Gx121	Ax11	Intergenic, -9 'A' substitution within possible RBS for PE family protein, PE15
**Rv1819c**	***bacA***	2063079	insertion	-	-	-	-	**C**	**C**	**C**	**C**	-x173	-x135	-x38	-x37 Cx2	Cx101 -x2	Cx33	Cx125 -x5	Cx10	Frame shift, 'G' insertion in bacA, vitamin B12 transport ATP-binding protein, BacA
**Rv2043c**	***pncA***	2289151	substitution	T	**A**	**A**	**A**	**A**	**A**	**A**	**A**	Tx218 Ax1	Ax137	Ax37	Ax51	Ax132	Ax37	Ax147	Ax12	Amino acid substitution in pyrazinamidase/nicotinamidase PncA (I31F)
		2368904	insertion	-	-	-	CGG/-	-	-	-	-	-x275	-x252	-x28 CGGx8	CGGx51 -x15	-x218	-x32	-x177	-x1	Intergenic, non-persisting mutation
		2584951	substitution	C	C	C	C	C	C	C	G	Cx237	Cx187	Cx38	Cx36	Cx80 Gx1	Cx23	Cx144 Ax1	Gx20	Intergenic
**Rv2326c**		2597976	insertion	-	-	-	-	-	**G**	**-/G**	**G**	-x166	-x97	-x36	-x32	-x80 Gx9	Gx13	-x46 Gx37	Gx9 -x1	Frame shift, 'C' insertion, ABC transporter ATP-binding protein
		2610704	substitution	G	A	G	G	G	G	G	G	Gx239	Ax222 Gx70	Gx71	Gx68	Gx201	Gx63	Gx174 Ax1	Gx12	Intergenic, non-persisting mutation
**Rv2428**	***ahpC***	2726149	substitution	T	T	T	T	T	**C**	**T/C**	**C**	Tx279	Tx218	Tx59	Tx82	Tx128 Cx11	Cx44	Tx87 Cx81	Cx18	Intergenic, ahpC -44 T->C substitution in oxyR-ahpC region
**Rv3033**		3393491	substitution	A	A	**A/G**	**A/G**	**G**	**G**	**G**	**G**	Ax180 Gx2	Ax123 Cx1	Ax23 Gx8	Ax36 Gx3	Gx121 Tx1	Gx27 Ax1	Gx111	Gx16	Amino acid substitution in hypothetical protein (S38G)
**Rv3200c**		3573526	substitution	C	C	C/T	T	C	C	C	C	Cx163	Cx109	Cx24 Tx10	Tx28 Cx5	Cx79	Cx27	Cx102	Cx13	Synonymous, non-persisting mutation, transmembrane cation transporter
**Rv3340**	***metC***	3726078	insertion	-	-	-	-	-	**GA**	**-/GA**	**GA**	-x235	-x159	-x40	-x44	-x91	GAx26	-x84 GAx12	GAx11	Intergenic, metC -49 'GA' insertion, methionine biosynthesis enzyme MetC
**Rv3854**	***ethA***	4327133	deletion	T	T	**T/-**	**T/-**	**-**	**-**	**-**	**-**	Tx217	Tx173	Tx31 -x11	Tx38 -x7	-x136 Gx1	-x49	-x164	-x10 Tx1	Frameshift, 'A' deletion, monooxygenase EthA
**Rv3854**	***ethA***	4327169	substitution	T	T/G	T/G	T/G	T	T	T	T	Tx225	Tx136 Gx33	Gx37 Tx13	Gx33 Tx6	Tx140 Gx1	Tx50	Tx164 Gx1	Tx9	Amino acid substitution in monooxygenase EthA (H102P)
		4403459	substitution	C	C	C	A/C	C	C	C	C	Cx270	Cx240 Ax1	Cx68 Ax1	Ax82 Cx13	Cx183 Ax2	Cx54 Ax1	Cx183	Cx9	Intergenic, non-persisting mutation
**Rv3919c**	***gidB***	4407928	substitution	T	T	T	T	T	G	T	G	Tx273	Tx211 Cx1	Tx49	Tx68	Tx168	Gx61 Tx3	Tx225 Cx1	Gx6	Amino acid substitution in rRNA small subunit methyltransferase, GidB (E92A)

Notes: Bases highlighted in red type face and bold border indicates persisting mutation; "A/C" notation indicates ambiguous base call at that position; "-" indicates deletion; '*' indicates cluster counted as a single mutation event
